# Transactions of the Kentucky State Medical Society

**Published:** 1877-11

**Authors:** 


*Transactions of the Kentucky State Medical Society. Twenty-Second Annual Convention. Held at Louisville, April, 3d, 4th and 5th,* 1877. John P. Morton & Co., Louisville, Ky.

Many papers of interest were presented before the society among which Chole-Lithiasis by Dr. John A. Octerlomy, Sterility and its treatment by W. H. Nathen, M. D., and Infantile Therapeutics by J. A. Larrabee are especially to be noticed. They are well written and modern. The following papers will be found above the average: Anæsthesia in Parturition by J. P. Yandell, M. D.; Pathology and Treatment of Sprains by R. O. Cowling, M. D. Report on Dermatology by J. P. Yandell, Jr., M. D. Physiological Therapeutics in fever by Jonn L. Cook, M. D.; and Deafness and some of its causes by M. F. Coomes, M. D.

The typography of the volume is excellent and does credit to the editing committee. The presidents address upon medical progress is refreshing in these times of lax professional practices. He sees in the near future a time when no one will be allowed to practice until he shall have shown himself competent. It can not, however, be accomplished in the manner he advises, until public opinion is changed, since the common people seem to favor charlatanism. C.

